# Decoding Hydrogel Porosity: Advancing the Structural Analysis of Hydrogels for Biomedical Applications

**DOI:** 10.1002/adhm.202500658

**Published:** 2025-06-17

**Authors:** M. A. Kristine Tolentino, Eric Y. Du, Giulia Silvani, Elvis Pandzic, Kristopher A. Kilian, J. Justin Gooding

**Affiliations:** ^1^ School of Chemistry and Australian Centre of NanoMedicine University of New South Wales Sydney NSW 2052 Australia; ^2^ School of Materials Science and Engineering University of New South Wales Sydney NSW 2052 Australia; ^3^ Katharina Gaus Light Microscopy Facility Mark Wainwright Analytical Centre University of New South Wales Sydney NSW 2052 Australia

**Keywords:** convex hull algorithm, diffusion, hydrogel structure, packing algorithm, particle tracking, porosity characterization

## Abstract

Hydrogels are essential biomaterials for biomedical applications, valued for their tunable properties and biocompatibility. A key feature influencing their function is porosity, which governs transport properties. Cryogenic scanning electron microscopy (cryo‐SEM) is widely used to directly characterize porosity, but may introduce structural artifacts. Accurately characterizing the porosity of a hydrogel in its native state remains a challenge. Here, we characterized the hydrogel porosity in its native state using particle tracking assay and compared the results with cryo‐SEM in polyethylene glycol (PEG) hydrogels. Both methods revealed the presence of micropores in PEG, likely arising from defects during polymerization. The equilibrium swelling assay showed nanoscale mesh sizes between polymer chains, distinct from the micron‐scale pores. To overcome conventional limitations, we developed a novel three‐dimensional (3D) pore reconstruction approach by leveraging the convex hull algorithm. The method enabled measurement of pore volume, surface area, sphericity, and size distribution. We found that cryo‐SEM underestimates pore diameters due to the two‐dimensional (2D) depiction, but after the 2D‐to‐3D conversion, remarkably similar pore dimensions are obtained. By advancing porosity analysis, this work provides insights for tailoring hydrogels to optimize interactions with cells, biomolecules, and therapeutic agents, opening avenues in drug delivery, tissue engineering, and other biomedical applications.

## Graphical abstract

Porosity governs transport mechanics in hydrogels, yet characterization in its native state remains challenging. This work harnesses particle tracking to uncover three‐dimensional pore geometric features in polyethylene glycol hydrogels. Measurements in the native state reveal pores characteristics are comparable to those obtained by cryogenic scanning electron microscopy. The approach yields previously inaccessible pore metrics in the native state, offering new possibilities for hydrogel design.

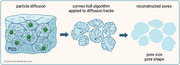

## 1 Introduction

Soft structures in nature that are made up of water‐swollen networks of biopolymers are classified as hydrogels. Ubiquitous in living systems, these natural hydrogels nurture biology. However, their properties are difficult to control, thereby limiting their use as components in technology. This challenge has driven the development of synthetic analogs, where the chemistry, architecture, and mechanical properties can be precisely tuned.^[^ ^1^, ^2^ ^]^ Such advancements make synthetic hydrogels indispensable tools for understanding how three‐dimensional (3D) environments influence biological processes. For instance, this capability to control variables critical for mimicking tissues revealed how the stiffness of the microenvironment where cells reside impacts proliferation,^[^ ^3^ ^]^ migration,^[^ ^4^ ^]^ and differentiation.^[^ ^5^ ^]^ However, changes in stiffness are often associated with changes in porosity, which can also influence cellular behaviors. The porosity of a hydrogel directly influences its mechanical and transport properties,and thus, its interaction with cells and biomolecules.^[^ ^6^, ^7^ ^]^ For example, porosity governs nutrient transport,^[^ ^8^ ^]^ cell infiltration,^[^ ^9^ ^]^ and tissue regeneration.^[^ ^10^ ^]^ Moreover, it may also facilitate crosstalk between cells and protein tethering.^[^ ^11^, ^12^ ^]^ In drug delivery systems, porosity controls the release profile of therapeutic agents.^[^ ^13^, ^14^ ^]^ Therefore, it is crucial to develop methods to accurately track the hydrogel porosity.

Hydrogels are comprised of nanoscale networks formed by polymer linkages. The spaces between these polymer linkages, referred to as mesh, are crucial in determining a range of hydrogel's properties. Larger spaces that arise from a variety of factors during polymerization and gelation are known as pores.^[^ ^15^ ^]^ Although the terms “mesh” and “pores” are often used interchangeably to describe hydrogel porosity, they differ significantly in scale and function. Nanoscale mesh primarily regulates the passive diffusion of small molecules, such as biomolecules, metabolites, and therapeutic drugs.^[^ ^13^ ^]^ In contrast, micron‐scale pores affect cell migration, as the size of the nucleus constrains a cell's ability to traverse the hydrogel matrix.^[^ ^9^, ^16^ ^]^ Understanding these distinctions is critical for tailoring hydrogel design to specific biomedical applications.

Various techniques have been developed to characterize hydrogel “porosity” and/or mesh. Each technique comes with its own strengths and limitations. An equilibrium swelling assay is a commonly used method to indirectly measure hydrogel mesh size using the Flory–Rehner equation, which links the swelling behavior of the hydrogel to its network structure.^[^ ^17^ ^]^ This assay gives an overall average mesh size, rather than a distribution of mesh size. Light scattering techniques, particularly small‐angle neutron scattering (SANS) and small‐angle X‐ray scattering (SAXS) have been utilized to measure the mesh and pore sizes of hydrogels.^[^ ^18^, ^19^ ^]^ These techniques relate the internal spacings within the hydrogel network to the intensity of scattered radiation as it passes through the sample. These techniques can probe across a broad range, from sub‐nanometre to sub‐micron scales. Micropore imaging of hydrogels has been achieved using confocal microscopy on gels that have been fluorescently labeled.^[^ ^18^, ^20^ ^]^ Chord length analysis, a well‐established data analysis method for quantifying porosity, can be applied to fluorescently labeled hydrogels by measuring segment lengths from randomly oriented lines crossing phase boundaries. The particle tracking assay, based on diffusion, is a technique that characterizes actual porosity with the hydrogel in its native state without the requirement for chemical modification. This technique involves embedding fluorescent particles within the hydrogel and tracking their motion over time. The movement of these particles is influenced by the hydrogel's pore size and the diffusion properties of the particles through the material.^[^ ^18^, ^21^ ^]^ It is commonly used in micro‐rheology to determine local viscoelastic properties. Furthermore, this technique can measure real‐time changes in a hydrogel with a dynamic structure.^[^ ^21^ ^]^ For example, measuring changes in hydrogel porosity during cell‐mediated remodeling.^[^ ^22^ ^]^ The limitation of the technique is it can only monitor pore sizes of the hydrogel that are larger than the trackable particle size. Another technique that can assess the hydrogel porosity in its native state is atomic force microscopy (AFM). AFM provides high‐resolution mapping of hydrogel surface topography, enabling the visualization of pore openings under hydrated conditions.^[^ ^23^ ^]^ AFM can also measure local stiffness simultaneously with surface topography. However, AFM is limited to surface‐level imaging and small scan areas, which may not reflect the full internal pore structure.^[^ ^24^ ^]^

Electron microscopy techniques offer direct visualization of hydrogel pore structures. Environmental scanning electron microscopy is an emerging approach that allows imaging of hydrogels in a partially hydrated state. However, it may not be suitable for all hydrogels. For example, this technique showed limited success in producing a clear microstructure of hydrated collagen and polyglycerol methacrylate hydrogel.^[^ ^25^, ^26^ ^]^ To bridge this gap, cryogenic scanning electron microscopy (cryo‐SEM) has become a powerful alternative as it can provide high‐resolution images of the hydrogel's morphology, allowing for direct observation of pore sizes, shapes, and distribution. However, cryo‐SEM imaging requires snap freezing of the water in the hydrogel, which can distort its native structure.^[^ ^27^ ^]^ This has become a growing concern for scientists wishing to characterize the porosity of their hydrogels.

To address this concern, we sought to develop a method capable of analyzing hydrogels in their native state, without the artifacts introduced by drying or freezing. Specifically, we aimed to evaluate the reliability of the pore sizes acquired via SEM imaging by comparing them to the particle tracking‐based approach, which preserves the native structure of the gel during analysis. Combining the results from these assays, we elucidated the structure of polyethylene glycol (PEG) hydrogel, a widely used extracellular matrix mimic.^[^ ^4^, ^28^, ^29^, ^30^, ^31^ ^]^ While particle tracking has previously been used to estimate average pore size, our approach introduces a novel method for analyzing diffusion data that quantify pore dimensions at the single‐pore level rather than a bulk average. To gain a more comprehensive understanding of the hydrogel structure, we leveraged a tool from computational geometry, the so‐called “convex hull” algorithm,^[^ ^32^, ^33^, ^34^, ^35^, ^36^ ^]^ that translates ensembles of particle trajectories into an outline of the hydrogel pore. By applying this algorithm to the particle tracking data, we reconstructed the hydrogel's internal structure and quantified critical metrics, such as the distribution of pore volume, surface area, diameter, and sphericity. These metrics are relevant for characterizing hydrogels used in various applications. For example, pore volume can indicate the space available for cells or therapeutic payloads, while pore surface area can serve as a measure of the accessible bioactive molecule in an entrapped cell. The pore diameter is the typically used metric to characterize pore size, and its low degree of dimension allows comparison of pores measured in two‐dimension (2D) and 3D. Sphericity can reveal how cell morphology adapts to the pores or how the pores adapt to the cells. Finally, using the 3D pores from the particle tracking assay, we simulated 2D images of the pores and their distributions to show how the particle tracking assay with convex hull analysis can provide analogous images to those obtained from SEM.

## 2 Results and Discussion

### 2.1 Micropores in Hydrogels Arise From the Defects in Mesh Formation

**Figure 1:**
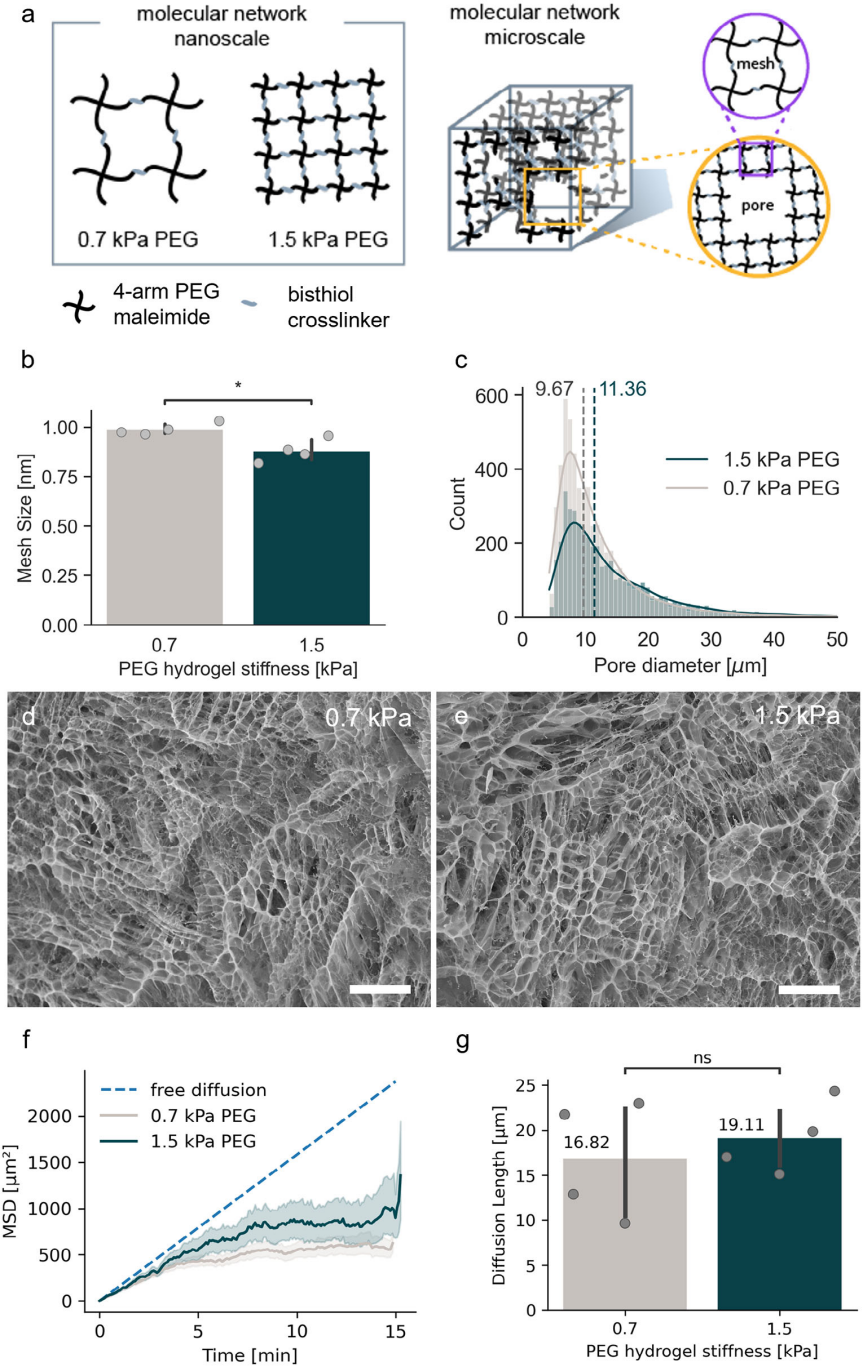
a) Schematic of the nanoscale and microscale molecular networks of PEG hydrogels in nanoscale and microscale. b) Mesh size of PEG hydrogels determined by a swelling assay based on Flory–Rehner equilibrium swelling theory. c) The pore diameter distribution of PEG hydrogels with median values of 9.67 and 11.36 µm for 0.7 and 1.5 kPa PEG, respectively. Pore data pooled from three samples for each hydrogel determined from the cryo‐SEM images of the PEG hydrogels: d) 0.7 kPa, and e) 1.5 kPa. The scale bar is 100 µm long. f) Mean‐squared displacement (MSD) curves showing the confined motion of the beads (n ≥ 1000) inside PEG hydrogels (*n* = 3) as compared to the linear behavior of simulated free diffusion. g) Diffusion length from the MSD curves in (f) as estimated pore sizes for 0.7 and 1.5 kPa PEG. T‐test: ^*^ indicates 0.01 < *p* ≤ 0.05, ns indicates not significant.

The chemical strategy we employed to control the stiffness of hydrogels involved varying the molecular weights of the polymer. This strategy inevitably altered the spacing between the crosslinked polymer units within the hydrogel. The 4‐arm PEG polymers with molecular weights of 10 and 20 kDa correspond to shorter and longer arm lengths, respectively. We crosslinked these 4‐arm PEG polymers via the maleimide‐thiol click reaction to create a low (0.7 kPa) and a high (1.5 kPa) crosslinking density hydrogels from the 20 and 10 kDa polymers, respectively. Theoretically, longer PEG‐arms result in larger spacings between crosslinked polymers than shorter arms (**Figure** **1**a), hence larger mesh size. We then characterized the spacings in these hydrogels using the widely used methods: equilibrium swelling assay and SEM imaging. Since stiffness is the controlled parameter and reflects the changes in crosslinking density, we present its values alongside mesh and pore sizes to illustrate the structural changes with varying mechanical properties.

Using the equilibrium swelling assay, we determined that the mesh size of the 0.7 kPa PEG hydrogel (0.99 nm) was larger than that of the 1.5 kPa PEG hydrogel (0.88 nm) as shown in Figure 1b. This result is consistent with the chemical design described above, illustrated in the nanoscale molecular network in Figure 1a. However, we achieved vastly different values using SEM measuring 9.67 µm for the 0.7 kPa hydrogel and 11.36 µm for the 1.5 kPa hydrogel (Figure 1c). These values were derived from SEM images, with representative images shown in Figure 1d,e. These SEM images show the pores, not the mesh of the hydrogel. The longest diameter for each pore was reported as the pore diameter. Notably, the pore diameters from the SEM images are in micron scale, which is three orders of magnitude different from the nanoscale values from the equilibrium swelling assay. While we acknowledged the concern that the sample preparation step for the cryo‐SEM may introduce artifacts to the samples,^[^ ^23^, ^26^ ^]^ we then employed particle tracking assay to confirm the presence of the micropores in the hydrogel as shown in our previous work.^[^ ^28^ ^]^ This method enables us to probe the micropores of the hydrogels in their native state. To ensure an accurate representation of pore accessibility, particle size selection was guided by testing the diffusion behavior of particles with diameters of 0.2, 0.5, 1, 2, 10 µm. Particles at the smallest size (0.2 µm) exhibited rapid diffusion, making them difficult to track reliably, while the largest (10 µm) were unable to diffuse freely. Based on these observations, intermediate sizes (0.5–2 µm) were deemed optimal for effective tracking and probing of micropore structures.

We characterized the diffusion behavior of 1 µm fluorescent beads (FluoSpheres) encapsulated in the hydrogels. The mean squared displacement (MSD) curves from the particle tracking assay are shown in Figure 1f. These MSD curves are derived from Equation (6). The simulated MSD curve of freely diffusing microbeads which were 1 µm in diameter was computationally generated based on the Stokes–Einstein equation (Equation 9) using the following variables that match the experimental conditions: T = 300 K, η = 0.001 Pa·s, r = 0.5 × 10^−6^ m. A linear increase in MSD of the freely diffusing beads graphically describes that moving beads continuously gained distance from their starting point over time. Whereas the moving beads encapsulated in PEG hydrogels reached a plateau in their MSD curves, these empirical data indicate that there is a maximum distance the beads can reach relative to their starting point inside the hydrogels. The maximum distance is attributed to the confined diffusion within the pores of the hydrogels. Since the pore size limits the MSD, this maximum distance is a function of the pore dimension. The beads were 1 µm in diameter and primarily diffused in pores greater than 1 µm in diameter. As such the movement of the beads indicates the presence of pores in the hydrogels that are significantly greater than 1 µm in size which is consistent with the length scale obtained from the cryo‐SEM images.

To estimate pore size, we calculated the diffusion length from the plateau in the MSD curves (Equation 7), as diffusion length is directly proportional to the average pore size. This was done by fitting a linear model along the plateau region of the MSD curve and extrapolating it to time zero (Figure S1, Supporting Information). The square root of the corresponding MSD value (y‐intercept) at time zero was then taken as the diffusion length. The calculated diffusion lengths for 0.7 and 1.5 kPa hydrogels are 17 (± 7) µm and 19 (± 4) µm, respectively, as shown in Figure 1g. These values are of similar magnitude and not statistically different (P = 0.63), although somewhat larger than the pore diameters measured from SEM, which are 9.67 µm for 0.7 kPa hydrogel and 11.36 µm for 1.5 kPa hydrogel, as shown in Figure 1c.

Notably, Figure 1f shows that the MSD curve of the 0.7 kPa PEG plateaued at lower values than that of the 1.5 kPa PEG, indicating that the pores in 0.7 kPa PEG are smaller than in 1.5 kPa PEG. From the MSD data, we determined the diffusion exponent denoted as *n* in Equation (8) (*MSD*  =  6*Dt^n^*) to describe the level of confinement.^[^ ^18^ ^]^ The slope of the linear equation, when log_e_(MSD) versus log_e_(t) is plotted, refers to the diffusion exponent. The particles embedded in 0.7 kPa PEG hydrogel have a higher diffusion exponent (0.41  ± 0.04) than the particles embedded in 1.5 kPa PEG hydrogel (0.35 ± 0.06). Moreover, both diffusion exponents are less than 1 which indicates that the particles undergo sub‐diffusive behavior in restricted regions. Meanwhile, the simulated diffusion exponent of the freely diffusing beads is equal to 1, which refers to the free diffusion behavior. Overall, the resulting MSD curves from the particle tracking assay in Figure 1f corroborate the pore measurements from the cryo‐SEM images that the pore sizes in the 0.7 kPa PEG are smaller than the 1.5 kPa PEG. Since particle tracking assay enables probing of micropores in the native state of the samples, this auxiliary measurement shows that the presence of micropores in the samples is not exclusively artifacts due to sample preparation from the SEM imaging. The presence of micropores was further confirmed through the infiltration of 1 µm fluorescent particles (Figure S2, Supporting Information). Additionally, we have previously demonstrated the migration of cytotoxic T lymphocytes (≈10 µm) within similar peptide‐crosslinked PEG hydrogels.^[^ ^28^ ^]^ Since T lymphocytes are known to migrate through porous collagen scaffolds by a proteolytically independent mechanism,^[^ ^37^ ^]^ the observed micron‐scale pores in this study provide a plausible explanation for how these cells can navigate through what is conventionally considered a nanoporous matrix. With both the nanoscale and microscale pore values, we infer that the micropores may be due to defects in mesh formation as depicted in the microscale molecular network in Figure 1a.

While 4‐arm PEG hydrogels are often referred to as “ideal polymer networks”,^[^ ^38^, ^39^, ^40^ ^]^ the incorporation of asymmetric peptide crosslinkers such as dicysteine MMP degradable sequences (Figure S3, Supporting Information) introduces geometric mismatch that inevitably leads to inhomogeneity like the micron‐scale pores observed in our study. These defects are likely compounded by non‐uniform crosslinking due to the rapid gelation afforded with Michael‐type addition reactions. The presence of micropores has been reported previously in other PEG‐based systems.^[^ ^41^ ^]^ A degree of microporosity is desirable for our applications because it mimics the hierarchical structure of native tissue matrices, which contain both nano‐ and microscale features.^[^ ^42^ ^]^

Due to its non‐destructive sample preparation, the particle tracking assay is an appealing approach to measuring the microporosity of hydrogels. While the assay provides an estimate of the pore sizes from the MSD curves, it does not offer information about the actual pore size and its distribution. This limitation led us to ask whether we could extend the particle tracking assay to generate a distribution of pore sizes and describe the hydrogel porosity more comprehensively. Next, we demonstrate how diffusion data can be used in a novel way to probe additional information about the microstructure of hydrogels beyond the diffusion length as an estimate for pore size.

### 2.2 Particle Diffusing within a Micropore can Probe the Geometric Properties of the Micropore

**Figure 2:**
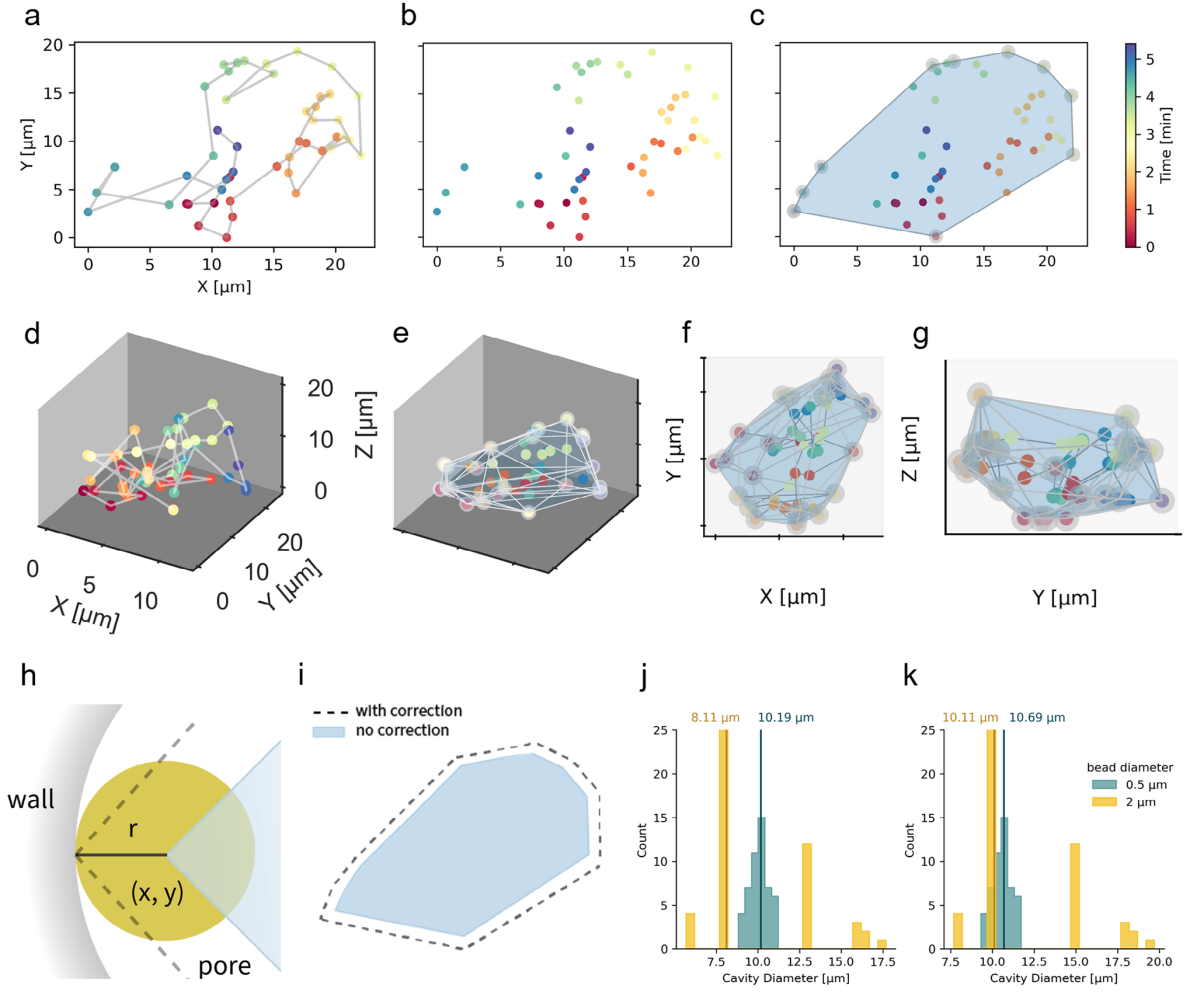
The convex hull algorithm enables geometric mapping of hydrogel geometry. The time stamp for each coordinate for a‐g is depicted in the color bar on the right of c. a) 2D tracks of particle diffusion. b) 2D tracks were simplified to position coordinates and the convex hull algorithm was applied. c) Visualized result of the convex hull algorithm applied to b. The position coordinates identified as vertices are highlighted in gray circles. The resulting 2D shape is shown in blue from which the pore dimensions can be extracted. d–g) The convex hull algorithm can also be applied to 3D position coordinates to generate a 3D shape. f) The gray lines show the edges of the faces of the 3D shape. (g) The side view perspective of f along the *yz* plane. h) Illustration of an identified vertex at position *x, y* which is the center of the yellow bead with radius *r*. The dashed gray lines are the better approximation of the boundary of the pore. i) Representative illustration of the 2D shape with correction (gray dashed line) and without correction (blue filled shape). Histogram of the cavity diameters determined using two different bead sizes j) without correction and k) with correction. The yellow and blue vertical lines show the median cavity diameter for the 2 and 0.5 µm bead diameters, respectively.

The motion of the particles in space is observed and tracked using a microscope and tracking algorithm. The representative tracks are shown in **Figure** **2**a for movement along a 2D plane and in Figure 2d for movement within a 3D space. When diffusion occurs in a micropore, the extent of particle movement is bound by the walls of the micropore. Observing and recording these repeated collisions of a particle against the wall in different parts of the micropore enables us to estimate the geometric information of the micropore. This is achieved by disregarding the time dimension of the track (Figure 2b) and connecting their outermost position coordinates. We can then create a structure that represents the solid shape of the micropore (Figure 2c,e–g). In other words, this involves generating the smallest convex shape that encloses a set of points, a mathematical concept known as the convex hull. To construct this structure and extract geometric information from our particle tracking data, we borrowed a tool from computational geometry known as the convex hull algorithm. The convex hull algorithm was implemented to the position coordinates of the tracks using python and its SciPy library.^[^ ^35^ ^]^ The resulting structures can then be used to calculate geometric properties such as volume, diameter, and shape that can describe the microstructures of a hydrogel. From here on, we refer to these computationally generated solid structures as geometric models.

### 2.3 The Dimensions of the Cavity can be Quantified by Applying the Convex Hull Algorithm to the Position Coordinates of the Tracked Particle

We encapsulated the polystyrene‐based particles with diameters of 0.5 and 2 µm in the same 0.7 kPa PEG hydrogel. We observed the particle diffusion in the same small area to determine whether the particle size influences the calculated geometric models of the micropore. The convex hull algorithm available in the SciPy library outputs the vertices of the geometric models from the position coordinates. These vertices were used to calculate the distance between all the vertices using Equation (10) and, the longest distance (also known as Feret's diameter) in a geometric model is reported as the diameter of the micropore. The diameters of the micropores of the hydrogels are shown in Figure 2j. Here, we observed a difference in the measured diameters as a function of particle size; the pores measured using 0.5 µm beads have a median diameter of 10 (± 1) µm whereas the median diameter using 2 µm beads is 8 (± 3) µm. This difference is attributed to the computational assignment of the particle's position coordinates to its centroid, rather than the edge of the particle which is in contact with the wall of the micropore (Figure 2h). This mismatch extends to all sides of the structure (Figure 2i). Thus, the calculated diameter can be corrected by adding the diameter of the particle used during the assay. Applying this correction results in better precision of the measured diameters with measured median diameters of 11 (± 1) µm and 10 (± 3) µm for 0.5 and 2 µm beads, respectively (Figure 2k).

Next, we tested the validity of the measured values we obtained from the particle tracking assay. To achieve that, we created cavities with known dimensions.

### 2.4 Geometric Models Accurately Determine Micropore Diameters

**Figure 3:**
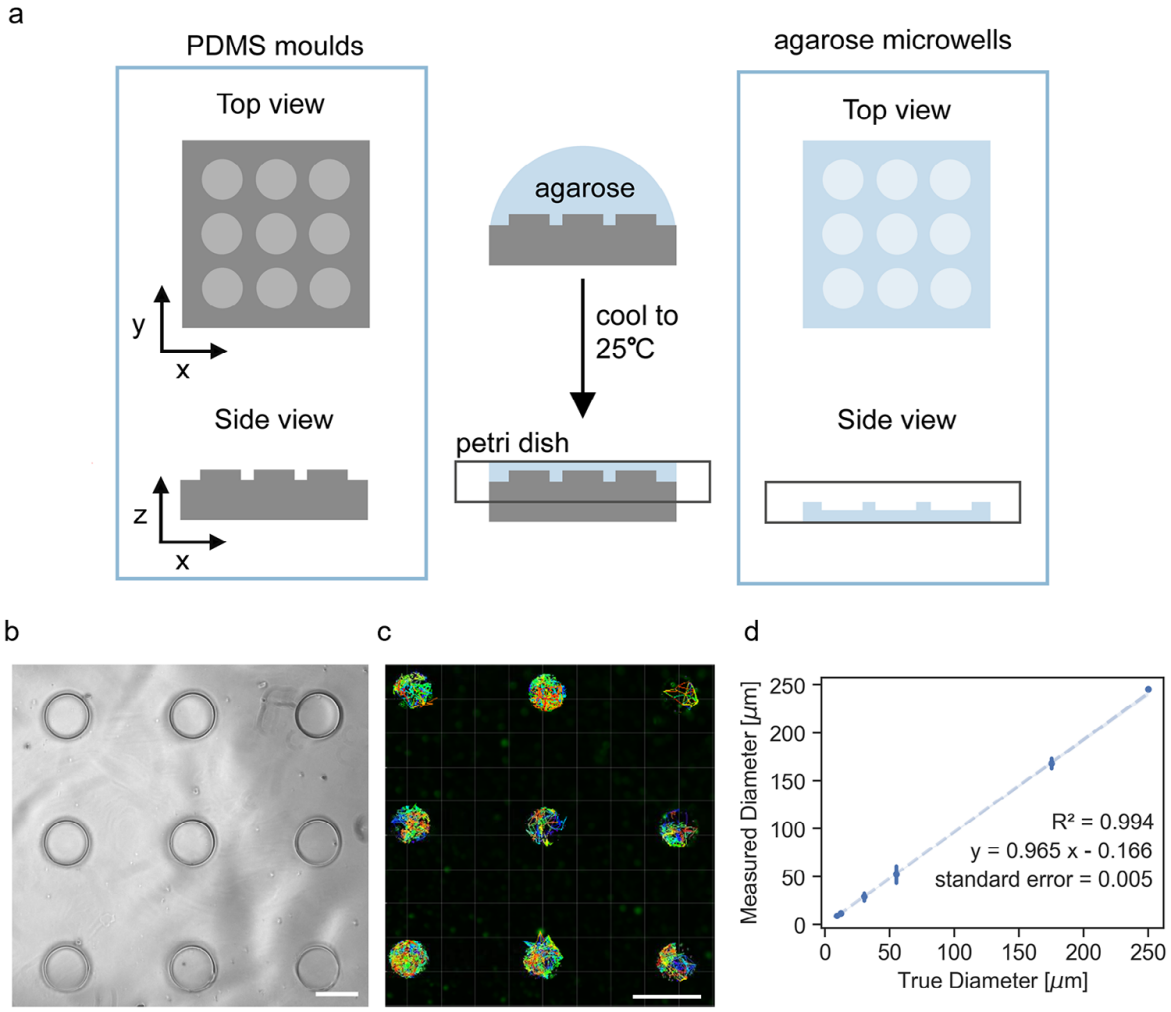
Micro‐structured models of confined volumes show reliable determination of pore diameters. a) Schematic for creating microcavity with defined dimensions. Hot liquid agarose was poured onto the polydimethylsiloxane (PDMS) molds to fabricate agarose microwells. b) Representative image of the resulting agarose 250 µm microwells (Scale bar = 250 µm). c) Representative result of particle tracking of fluorescent beads in 55 µm microwells with defined dimensions (Scale bar = 100 µm). d) Linear correlation of the measured diameters using convex hull particle diffusion analysis against the true fabricated diameter of the microwells (*n* = 4–52).

We created agarose microwells with diameters of 9, 12, 30, 55, 175, and 250 µm using polydimethylsiloxane (PDMS) molds (**Figure** **3**a). The resulting microwells assumed the shape and size of the mold (Figure 3b). Fluorescent particles were then drop‐casted to the microwells, and their diffusion was observed and tracked (Figure S4, Supporting Information). Figure 3c shows representative tracks from the image analysis. The convex hull algorithm was applied to the position coordinates of these tracks to calculate the measured diameter as described in the preceding section. The measured diameters from this analysis are correlated against the true diameter from the PDMS mold to validate the measurement from particle tracking. Figure 3d shows a sensitivity of 0.965 and a strong linear correlation (R^2^ = 0.991) with a low standard error of regression (0.005). These values indicate that the change in true diameter in the microwell causes the same change in the measured diameter using the particle tracking assay. Overall, Figure 3d establishes the reliability of the measurements obtained using particle tracking assay.

### 2.5 3D Micropore Geometry was Determined Using Particle Tracking Assay

**Figure 4:**
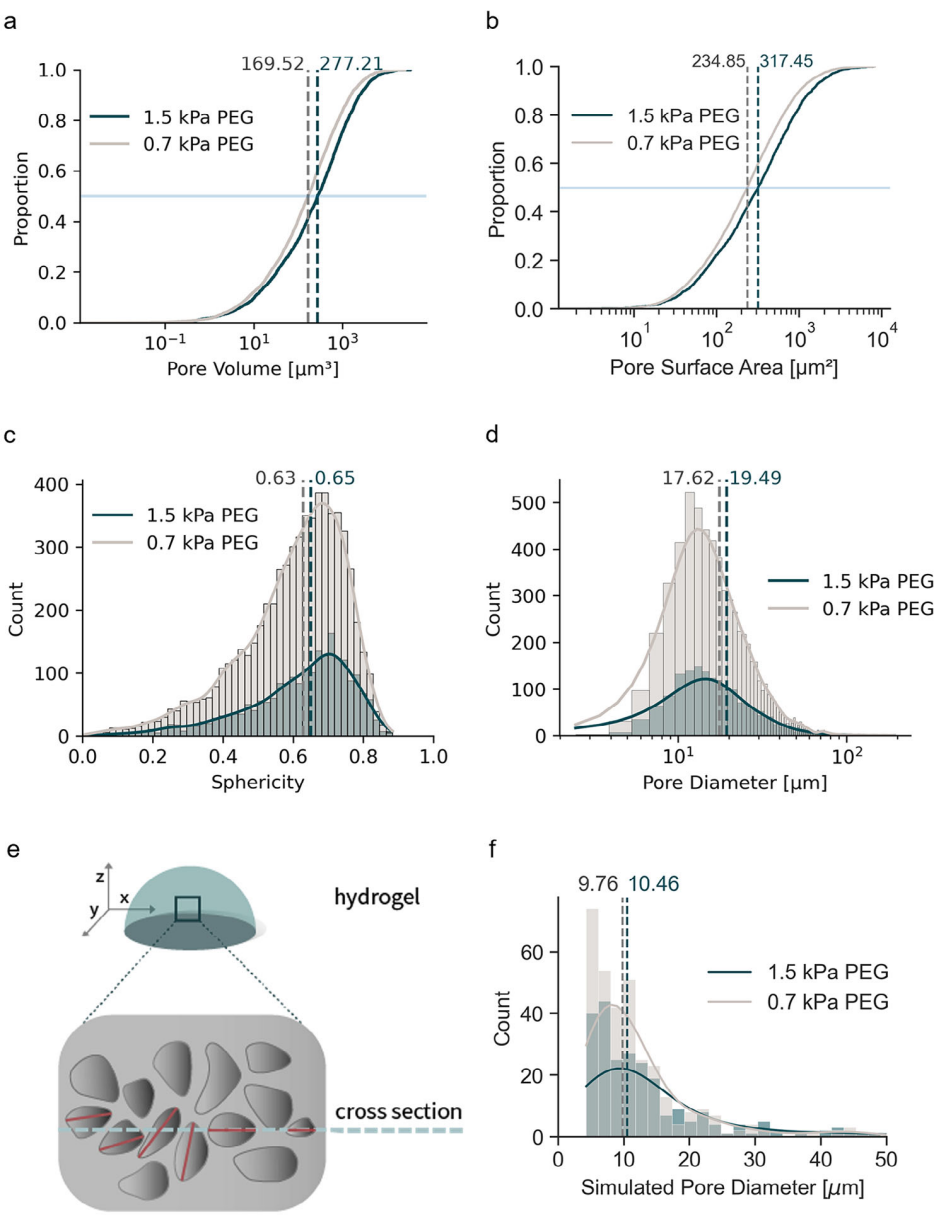
Measurement of pore dimensions of the PEG hydrogels using the convex hull algorithm: a) empirical cumulative distribution function of pore volume and b) pore surface area, histograms of c) sphericity, and d) pore diameter. Dashed lines of the same color legend represent the median values. Data was pooled from four samples of each PEG hydrogel. e) Schematic of a gel cross‐sectioned along the *xy* plane (blue dashed line) which may or may not overlap with the longest diameter of the 3D pores represented as red lines. f) Simulated SEM pore diameters derived from the empirical 3D pores which is comparable to the values obtained from the empirical SEM data in Figure 1c.

From the same data that generated the MSD curves in Figure 1f, we applied the convex hull algorithm and generated the geometric models, which refer to the computationally reconstructed 3D pores. Figure 1f shows that we collected sufficient diffusion data to trace the pores: the MSD curves of particles diffusing inside the hydrogels plateau after 5 min and we kept collecting diffusion data for the next 10 min. With this diffusion data, we measured the relevant dimensions to better elucidate the microstructure of the hydrogels such as pore volume, surface area, sphericity, and pore diameter (**Figure** **4**a–d). Pore volume and surface area were determined using the built‐in functions in the SciPy library. Then, sphericity was calculated from the pore volume and surface area using Equation (11).

The pore volume, surface area, and diameter of the 1.5 kPa PEG hydrogel are higher than that of 0.7 kPa PEG hydrogel (Figure 4a,b,d). The sphericity of the micropores in both hydrogels is similar. These measured values support the micropores from the SEM images (Figure 1c–e) and the MSD curve approximation (Figure 1f,g). Notably, the median pore diameters (17.62 µm and 19.49 µm) in Figure 4d and the mean diffusion lengths (16.82 and 19.11 µm) in Figure 1g are very close, indicating that diffusion length is a suitable estimate of the pore diameter.

Although we achieved a similar length scale value in micropore diameters from the SEM images and the geometric models, there is ≈10 µm deviation in the measured micropore diameters which is non‐trivial. We hypothesized that the difference in imaging dimension causes the deviation in the measured pores; the SEM images were in 2D while the geometric models were in 3D. During sample preparation for SEM, the hydrogel was cross‐sectioned to reveal the internal structure. The SEM images were taken from the cross‐sectioned plane which may or may not align with the longest diameter of its micropores in 3D as illustrated in Figure 4e.

To test our hypothesis, we used the reconstructed pores generated by the convex hull algorithm (3D geometric models) to simulate an image that approximates the information obtained from an SEM; that is a 2D projection showing the range of pore sizes and their distributions. In essence, the 3D geometric models were transformed into 2D geometric models. To achieve this, we removed the *z*‐coordinate from each vertex in the 3D geometric models, effectively flattening the structures to 2D. Subsequently, the convex hull algorithm was reapplied to the resulting *xy* coordinates of the vertices, generating planar 2D geometric models. A simplified illustration of this principle can be found in Figure S5 (Supporting Information). These 2D geometric models served as simulated pores as observed in SEM. The distribution of pore diameters of the simulated SEM pores in Figure 4f closely matched those of the empirical SEM pore diameters in Figure 1c, demonstrating the alignment of our computational pore geometry with SEM measurements. Furthermore, since we were able to simulate the empirical 2D SEM pores from our 3D geometric models measured in the native state of hydrogels, our data shows that at this length scale, SEM imaging provides an accurate depiction of 2D pore size. Next, we aimed to visualize the simulated 2D pores to gain a better understanding of their structural features.

### 2.6 Abstract Representations of the Flattened Geometric Models were Generated through Computational Image Reconstruction

**Figure 5:**
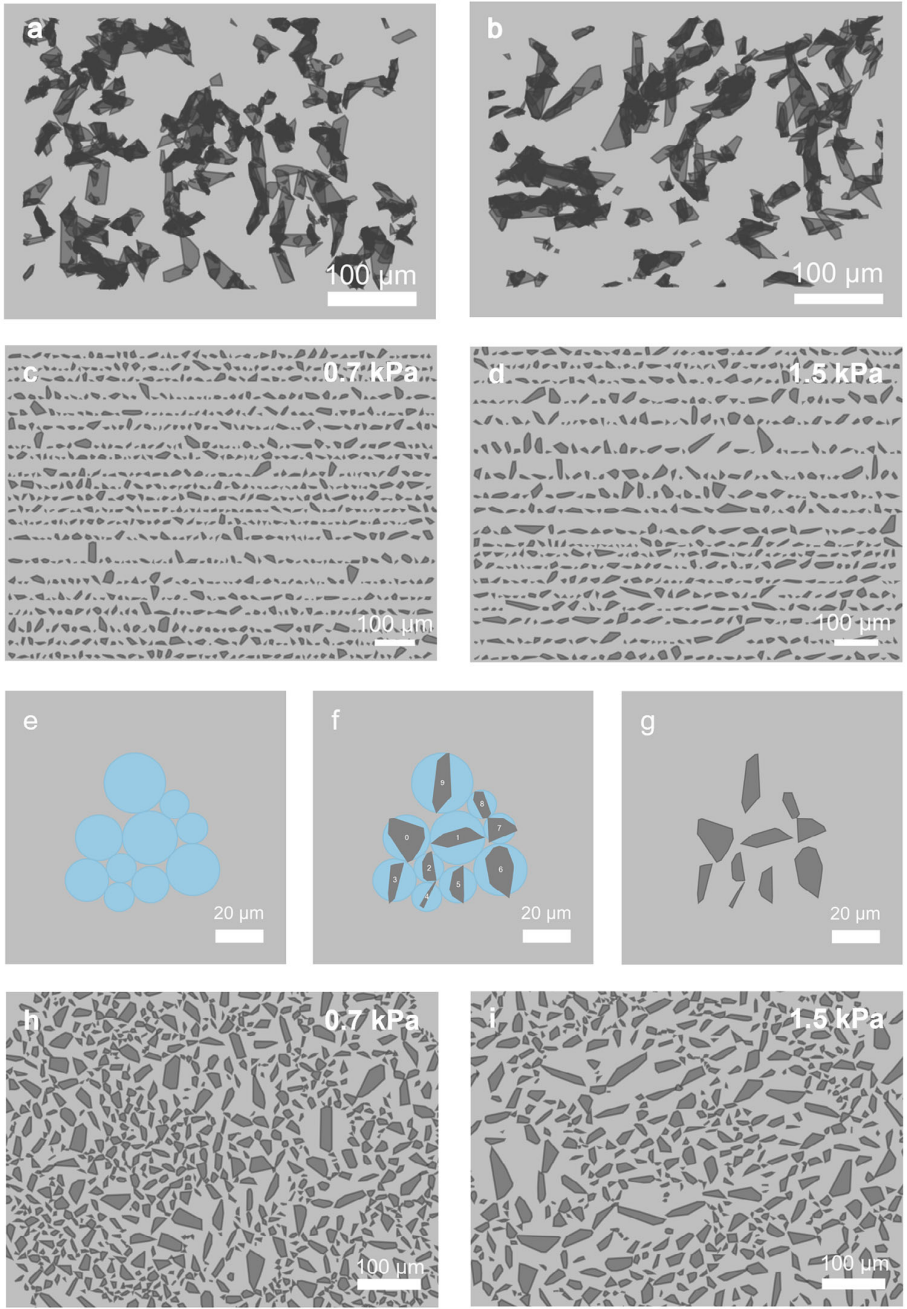
Reconstruction of pseudo‐2D pore images derived from the simulated 2D pore data. Reconstructed 2D pores from the flattened geometric models (a,b) as is, and (c,d) with a 10 µm gap between pores. e) Using the diameter of a subset of geometric models, circles were generated and fitted together using the circle packing algorithm. These circles serve as a placeholder for the flattened geometric models. f) The flattened geometric models were circumscribed to their corresponding circles. g) The model circles were removed. This packing method was scaled up to the data and used to reconstruct the pseudo‐2D pore images of h) 0.7 kPa and i) 1.5 kPa hydrogel.

We used the 2D geometric models that simulated the 2D diameters in Figure 4f to reconstruct pseudo‐2D pore images (**Figure** **5**). Plotting these models in their original positions reveals apparent spatial overlaps between the pores in both hydrogels (Figure 5a,b). This occurs because flattening pores from different *z*‐planes lose spatial resolution along the *z*‐axis, causing pores from different layers to appear as if they occupy the same *z*‐plane. As a result, any overlaps in their *xy* coordinates appear as overlaps in Figure 5a,b. This overlap makes it challenging to clearly discern the shape and size of the pores from the 2D geometric models.

To visualize the individual pores without overlaps, we adjusted the position of the pores to eliminate the overlaps while preserving their shapes, sizes, and spatial orientations. Following this principle, we employed two approaches: 1) plotting each pore with a 10 µm gap distance between them along the *x*‐axis (Figure 5c,d), and 2) plotting each pore with varying gap distances that maximizes their packing density (Figure 5h,i). Both approaches ensured that the pore's geometric features remained intact despite the loss of the original position.

Plotting each pore with a fixed gap distance as in Figure 5c,d is computationally straightforward as it is unnecessary to consider each pore's shape and orientation. However, this was not the case when we fit pores with varying gap distances to pack them tightly. The majority of the pores are irregular polygons, making the computational implementation significantly more complex. To achieve this, we utilized another tool from computational geometry known as the packing algorithm. We used the Python implementation (packcircles 0.14) developed by Wang et al.^[^ ^43^ ^]^ To prototype the image reconstruction, we selected a small subset of the data. The pore diameters of the 2D geometric models were used to create circles of matching size, which were then tightly packed using the circle packing algorithm (Figure 5e). These packed circles functioned as placeholders for the pores, with each circle's diameter corresponding to the pore it represented. The centroid of each pore was calculated using Equations (12) and (13). Then, each centroid was repositioned to match the coordinates of the center of the placeholder circle. Using the re‐positioned centroids as the reference points, the vertices of the pores were adjusted, resulting in pores circumscribed around their corresponding circles (Figure 5f). After that, the placeholder circles were removed to show the pseudo‐2D pore images (Figure 5g). This algorithm was subsequently scaled up to the entire dataset, generating Figures 5h,i. A subset of these packed 3D pores is also visualized in the form of rotating models (Movie S1, Supporting Information).

To validate our approach, we compared the distribution of pore diameters between the pseudo‐2D pores and the SEM‐derived pores using Mann–Whitney U test. The p‐values were 0.74 for 0.7 kPa hydrogel and 0.58 for 1.5 kPa hydrogel, indicating no significant difference between the size distributions of the pseudo‐2D pores and pore sizes determined from the 2D SEM images. These results confirm that the abstract representations resemble the structural features of the hydrogel pores captured in SEM imaging. These pseudo‐2D representations not only complement the quantitative metrics but also enable us to visualize the pore structures derived from the integrated particle tracking assay and convex hull algorithm, advancing our understanding of hydrogel porosity.

While our method provides a practical and accessible approach to visualize and quantify micropores in hydrogels, certain limitations remain. Pores smaller than the smallest particle size used in the assay may not be detected, potentially leading to an underestimation of finer‐scale porosity. This limitation can be addressed by complementing our method with SANS or SAXS, which are capable of probing shorter length scales. Additionally, factors that restrict diffusion such as particle adsorption onto the polymer network may impact the feasibility of particle tracking. Although this issue can be mitigated using the appropriate surface chemistries, currently available commercial particle probes offer limited chemical functionalities. Employing chemical strategies to modify the surface chemistry of the particles may help overcome this limitation. Future efforts may benefit from the development of a broader library of particle probes with tunable properties, as well as integrating complementary imaging techniques such as super‐resolution microscopy to enhance resolution and extend the detectable size range. These advancements would help improve the accuracy and versatility of the method, ultimately enabling a more comprehensive understanding of hydrogel microstructures in biomedical applications.

## 3 Conclusion

Our work shows that different techniques to describe porosity capture distinct structural features in PEG hydrogels. By consolidating the results from equilibrium swelling, cryo‐SEM imaging, and particle tracking assay, we characterized the 3D architecture of the multiscale pore network in PEG hydrogels. Notably, cryo‐SEM and particle tracking assay provide consistent information about the presence of micropores in these hydrogels. These micropores remained largely unaffected by varying the polymer molecular weight which, however, effectively tuned the nanopores (mesh). We infer that micropores are likely formed by defects during polymerization. Moreover, by extending the particle tracking analysis through the convex hull algorithm, we presented previously inaccessible metrics —such as pore volume, surface area, and sphericity— captured in the hydrogel's native state. Elucidating pore geometry is beneficial to understanding how hydrogels interact with cells, biomolecules, and therapeutic agents and could be key in engineering hydrogels for specific biomedical applications. Furthermore, a comparison between the real 2D SEM pores and the simulated 2D pores from the 3D geometric models revealed that 2D imaging (cryo‐SEM) tends to underestimate the real pore diameters. Although, the SEM images still provide an accurate depiction of 2D pores in PEG hydrogel. Overall, these findings highlight the importance of integrating multiple approaches to comprehensively characterize the porosity of PEG hydrogels.

## 4 Experimental Section

### Materials

FluoSpheres carboxylate‐modified microspheres with diameter sizes of 0.5, 1, and 2 µm (λ_ex/em_ = 505/515 nm, supplier: Invitrogen) were used as the fluorescent particles for the particle tracking assay. Dulbecco's phosphate‐buffered saline (DPBS, supplier: Gibco) was used as the buffer throughout the experiments. Microwells were fabricated using SYLGARD 184 silicone elastomer kit (supplier: Dow Chemicals) and agarose (supplier: Sigma–Aldrich). The components for the hydrogels were 4‐arm 10 kDa polyethylene glycol (PEG) maleimide (F38 and F119, supplier: Inventia Life Sciences) and bisthiol crosslinkers (F177 and F178, supplier: Inventia Life Sciences). All reagents were acquired from commercial vendors and used without further purification.

### Mesh Size Calculation

Mesh size was calculated using the Flory–Rehner equation according to previous literature.^[^ ^44^, ^45^ ^]^ The Swelling ratio was calculated for relaxed gels (*Q_mr_*) and swollen gels (*Q_ms_*) by preparing 200 mL of each gel which was weighed, allowed to swell for 24 h, and then weighed again. Relaxed gels refer to the gel in its initial state immediately after crosslinking, while swollen gels refer to the gel that has absorbed solvent and has reached swelling equilibrium. The samples were then lyophilized and weighed again. The wet weight and dry weight of each sample was applied to Equation (1):

(1) $$Mass\;swelling\;ratio=Q_{m}=\frac{m_{wet}-m_{lyophilised}}{m_{lyophilised}}$$

The volumetric swelling ratio (*Q_v_*) describes the increase in volume relative to the dry volume of the polymer network and can be derived from the mass swelling ratio (*Q_m_*). *Q_v_* was then calculated using the Equation (2):

(2) $$\;Q_{v}=1+\frac{\rho_{p}}{\rho_{s}}\left(Q_{m}-1\right)$$

where polymer density (*r_p_*) for PEG was taken as 1.125 g cm^−3^. Solvent density (*r_s_*) was taken as 1.011 g cm^−3^ for PBS. Relaxed polymer volume fraction (*υ_2r_*) and equilibrium polymer volume fraction (*υ_2s_*) was calculated using Equation (3):

(3) $$\upsilon =\frac{1}{Q_{v}}\;$$

The Flory–Rehner equation (Equation 4) was then applied to calculate the molecular weight between crosslinks (*M_c_*) where *M_c_* is the average molecular weight of the polymer prior to crosslinking, $\bar{\upsilon }$ is the specific volume of the polymer (taken as the reciprocal of density), *V_1_* was the molar volume of the solvent (18 mL mol^−1^ for water), and *X_1_* was the polymer‐solvent interaction (0.426 for PEG ^[^ ^46^ ^]^):

(4) $$\frac{1}{M_{c}}=\frac{2}{M_{n}}\;-\frac{\frac{\bar{\upsilon }}{V_{1}}\left[\ln\left(1-\upsilon_{2s}\right)+\upsilon_{2s}+X_{1}\upsilon_{2s}^{2}\right]}{\upsilon_{2r}\left\lfloor {\left(\frac{\upsilon_{2s}}{\upsilon_{2r}}\right)}^{\frac{1}{3}}-\frac{1}{2}\left(\frac{\upsilon_{2s}}{\upsilon_{2r}}\right)\right\rfloor }$$

Lastly, mesh size (ξ) was calculated using Equation (5), where *M_r_* is the molecular weight of the repeating unit, *l* is the bond length along the polymer backbone (0.15 nm for C─C bonds), and *C_n_* is the Flory characteristic ratio (4 for PEG ^[^ ^46^ ^]^):

(5) $$\xi =\upsilon_{2s}^{-\frac{1}{3}}\;l\sqrt{\frac{2C_{n}M_{c}}{M_{r}}}$$

### Scanning Electron Microscopy Imaging

The morphological structure of PEG hydrogels was imaged using JEOL JSM‐6490LV scanning electron microscope (SEM) as described previously.^[^ ^28^ ^]^ Briefly, the hydrogels were incubated in DPBS for 24 h. The sample holder was loaded with hydrogel with ≈0.5 mm of the hydrogel protruding from the base. Then, it was immersed in liquid nitrogen for 40–45 s, causing the hydrogel to rapidly freeze in its hydrated form and the protruding hydrogel was fractured to expose its cross‐section, revealing the internal hydrogel structure for imaging. The fractured sample was immediately placed inside the SEM chamber, vacuumed, and imaged within 5–10 min to minimize the sublimation of the frozen water in the hydrogel. The SEM micrographs were collected from a secondary electron signal at 15 kV. The pore diameters of the hydrogels from the micrographs were measured using ImageJ. The image was convolved with a 5 × 5 convolution kernel to highlight the fibers from the background. Pores with less than 10 µm^2^ area were excluded in the dataset upon visual inspection of the smallest detectable pores.

### Single‐Particle Tracking Assay in Microwells

The polydimethylsiloxane (PDMS) mold was manufactured by replica molding blending PDMS and crosslinker at a ratio of 10:1 and curing the degassed mixture in a SU‐8 master mold at 60 °C for a minimum of 2 h. The SU‐8 mold was specifically designed for the experiment using CAD software and realized with a soft lithography process. The design consists of circular shapes of different diameters (*x*, *y*, *z* microns) and a depth of 45 µm. After PDMS polymerization, PDMS was peeled off and used as a second mold to create microwells with varying diameters. Half a gram of agarose was added to 10 mL DPBS to achieve a 5% agarose solution. To fully dissolve the agarose in DPBS, the mixture was heated in a microwave oven for 10–15 s. The resulting hot viscous solution was transferred and kept onto a hot plate at 80 °C to keep the solution warm and avoid premature gel formation. The hot agarose solution (300–500 µL) was poured onto the top of the PDMS mold. A 35 mm petri dish was then immediately placed on top of the PDMS mold covered with hot agarose. The pressed hot agarose in the setup was cooled to room temperature for 5 min. Then, the PDMS mold was carefully peeled off from the petri dish. The resulting structure on the petri dish served as a microwells with known dimensions. After 24 h, 1 µm fluorescent particles diluted with DPBS (1:1000) were added to these microwells and particle diffusion was observed with Zeiss CellDiscoverer 7 900 using Plan‐Apochromat 20×/0.7 air objective (excitation wavelength = 470 nm, emission wavelength = 510–540 nm) with a pixel size of 0.46 µm. The images were collected every 5 s.

### Single‐Particle Tracking Assay in PEG Hydrogels

Fluorescent particles were mixed with the crosslinker (1:1000 volume ratio). In a 96‐well plate, 4‐arm PEG maleimide (5 µL) was carefully added onto the top of the bisthiol peptide crosslinker mixed with fluorescent particles (5 µL). The hydrogel was allowed to form for 5 min and DPBS (200 µL) was added to immerse the hydrogel. The resulting hydrogel with embedded particles was incubated for 24 h to complete swelling. Thereafter, particle diffusion was observed with Zeiss CellDiscoverer 7 900 using Plan‐Apochromat 20×/0.7 air objective (excitation wavelength = 470 nm, emission wavelength = 510–540 nm) with a pixel size of 0.46 µm. The 3D image stacks (total depth: 20 µm) were collected every 5 s with a z‐step size of 1 µm.

### Data and Statistical Analysis of Particle Tracking

Particle tracking was performed using Imaris 9.5.1 (Bitplane). The tracking algorithm used was Brownian motion with a maximum gap distance of 20 µm and a maximum gap size of 1 time point. For the tracked beads, mean squared displacement (MSD) was calculated from the position coordinates over time.^[^ ^47^ ^]^ The MSD at time *t* refers to the mean square of the displacement traveled by the beads relative to their starting point at time 0, normalized to bead count (Equation 6):

(6) $$MSD=\frac{1}{N}\sum_{i=1}^{N}\left({\left(x_{t}^{\left(i\right)}-x_{0}^{\left(i\right)}\right)}^{2}+{\left(y_{t}^{\left(i\right)}-y_{0}^{\left(i\right)}\right)}^{2}+{\left(z_{t}^{\left(i\right)}-z_{0}^{\left(i\right)}\right)}^{2}\right)$$

where *N* is the number of beads, *x*, *y*, and *z* are the position coordinates of the *i* ^th^ bead at time 0 or time *t*. From the MSD curves, the MSD value corresponding to the plateau was used to calculate the diffusion length as shown in Equation (7):

(7) $$Diffusion\;Length=\sqrt{MSD}$$

The 3D lag‐time dependence of the MSD curves^[^ ^18^ ^]^ was described by Equation (8):

(8) $$MSD=6Dt^{n}$$

where *D* refers to the effective diffusion coefficient and *n* refers to the diffusion exponent.

The diffusion coefficient can be calculated using the Stokes–Einstein equation as shown in Equation (9):

(9) $$D=\frac{k_{b}T}{6\pi \eta r}\;$$

where *D* refers to the diffusion coefficient, k_b_ refers to the Boltzmann constant (1.38 × 10^−23^ J·K^−1^), *T* refers to the temperature in Kelvin, *η* refers to the viscosity of the medium where the tracer diffuses, and *r* refers to the radius of the fluorescent particle (tracer).

Position coordinates and the MSD of the detected particles over time were analyzed in a Jupyter notebook (Python 3.11.5 with the following libraries: pandas 2.0.3, SciPy 1.11.1, statannot 0.2.3, Matplotlib 3.7.2, Seaborn 0.12.2). The Pandas library was used to handle and perform operations on position coordinates. The SciPy library was used to calculate geometric information. The statannot library was used to determine statistical significance between groups. The Matplotlib and Seaborn libraries were used to visualize data. The convex hull algorithm was exported from the Python library SciPy.^[^ ^35^ ^]^ Then, it was applied to the position coordinates grouped according to Track ID. Track ID was the unique identifier of a particle. The resulting volume, surface area, and vertices of the convex hull were collected. From these measurements, diameter, and sphericity were calculated. The diameter of the convex hull was based on the longest distance between the vertices in the convex hull and calculated using Equation (10).

(10) $$Diameter=\sqrt{{\left(x_{i}-x_{j}\right)}^{2}+{\left(y_{i}-y_{j}\right)}^{2}+{\left(z_{i}-z_{j}\right)}^{2}}$$

where *x, y, z* are position coordinates of a distinct particle; *i* and *j* refer to *i* ^th^ and *j* ^th^ time at which the coordinates were observed. Sphericity was calculated using Equation (11).

(11) $$\mathrm{Sphericity}=\;\frac{\pi^{1/3}{\left(6\times \mathrm{pore}\;\mathrm{volume}\right)}^{2/3}}{\mathrm{pore}\;\mathrm{surface}\;\mathrm{area}}$$

The custom‐made code to extract diameter, surface area, and volume from diffusion data using a convex hull algorithm is available at https://github.com/ktolen/geometric‐models. The code was designed for users of all skill levels, including those with little to no programming experience.

### Simulation of 2D SEM Pore Data from Empirical 3D Pores

The empirical 3D pores generated from the convex hull algorithm were randomly selected using a custom‐built code where the total volume of the pores for each gel sample (0.7 and 1.5 kPa) was equal or a little less than 300 000 µm^3^ to normalize the sample volume. These randomly selected 3D pores were transformed into 2D shapes by removing the *z* coordinate of each vertex of the 3D pore, effectively flattening them to an *xy* plane. These 2D shapes served as the simulated SEM pores. Then, the distance between each vertex in the simulated SEM pore was calculated. For each simulated pore, the longest distance was selected to represent the diameter of the SEM pore. The difference in image resolution between the cryo‐images and the fluorescence images was normalized by determining the smallest diameter in the cryo‐EM images and filtering the diameters smaller than the smallest diameter in cryo‐images out of the simulated data.

To create the abstract representation of these simulated pores, the centroid for each pore was calculated using Equations (12) and 13. These equations were used to calculate the centroid of a closed polygon.

(12) $$C_{x}=\frac{1}{6A}\;\sum_{i=0}^{n-1}\left(x_{i}+x_{i+1}\right)\left(x_{i}y_{i+1}-x_{i+1}y_{i}\right),$$

(13) $$C_{y}=\frac{1}{6A}\;\sum_{i=0}^{n-1}\left(y_{i}+y_{i+1}\right)\left(x_{i}y_{i+1}-x_{i+1}y_{i}\right),$$

where *C_x_* and *C_y_* refer to the coordinates of the centroid; *x* and *y* refer to the coordinates of the vertices. *A* refers to the pore area, *n* refers to the number of vertices, and *i* refers to the *i* ^th^ vertex. The sequence of the vertices for calculation should be done in a counterclockwise order with the first and last element being the same coordinates.

### Statistical Analysis

All numerical data and statistical analysis were conducted in Jupyter Notebook using Python (version 3.11.5). Data processing was performed with Pandas (version 2.0.3) and SciPy (version 1.11.1), which was also used to assess normality via the D'Agostino‐Pearson test. Statistical comparisons were carried out using the Statannot library (version 0.2.3), applying the unpaired *T*‐test or Mann–Whitney U test with Bonferroni correction. The statistical differences were determined based on the *p*‐values, where ^*^ indicates 0.01 < *p* value ≤ 0.05, and *ns* indicates not significant. All measurements were performed with at least three replicates. Data visualization was done using Matplotlib (version 3.7.2) and Seaborn (version 0.12.2).

## Conflict of Interest

The authors declare no conflict of interest.

## Supporting information



Supporting Information

Supplemental Movie 1

## Data Availability

The data that support the findings of this study are available from the corresponding author upon reasonable request.
