# Supplementary material for: Decoding Hydrogel Porosity: Advancing the Structural Analysis of Hydrogels for Biomedical Applications
Source: Adv Healthc Mater. 2025 Jun 17;14(22):2500658. doi: 10.1002/adhm.202500658 (PMC12391661; doi:10.1002/adhm.202500658)
Supplement: Supplementary file 1 — Supporting Information [file ADHM-14-0-s002.docx]

**Supplementary Information**

**Decoding hydrogel porosity: advancing the structural analysis of hydrogels for biomedical applications**

M.A. Kristine Tolentino^1^, Eric Y. Du^1^, Giulia Silvani^2^, Elvis Pandzic^3^, Kristopher A. Kilian^1,2*^, J. Justin Gooding^1*^

^1^ School of Chemistry and Australian Centre of NanoMedicine, University of New South Wales, Sydney, New South Wales 2052, Australia

^2^ School of Materials Science and Engineering, University of New South Wales, Sydney, New South Wales 2052, Australia

^3^ Katharina Gaus Light Microscopy Facility, Mark Wainwright Analytical Centre, University of New South Wales, Sydney, New South Wales 2052, Australia

*Corresponding author. Email: [justin.gooding@unsw.edu.au](mailto:justin.gooding@unsw.edu.au). [k.kilian@unsw.edu.au](mailto:k.kilian@unsw.edu.au)


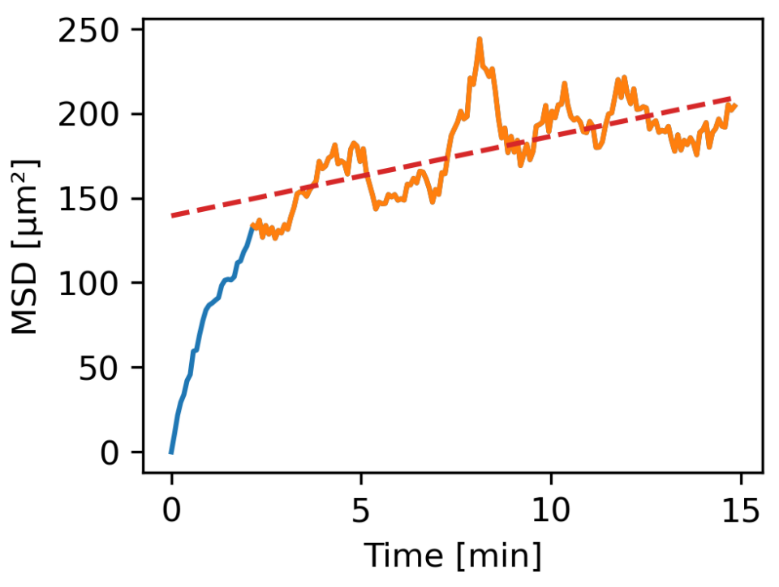


**Figure S1.** Representative plot illustrating the estimation of pore size from the diffusion length.


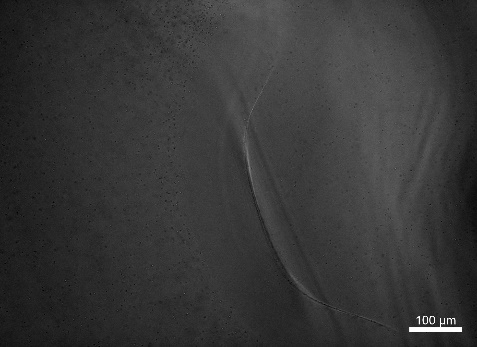

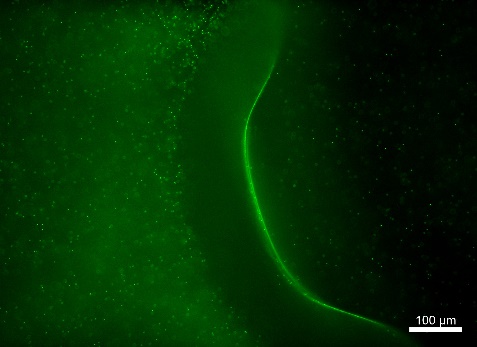

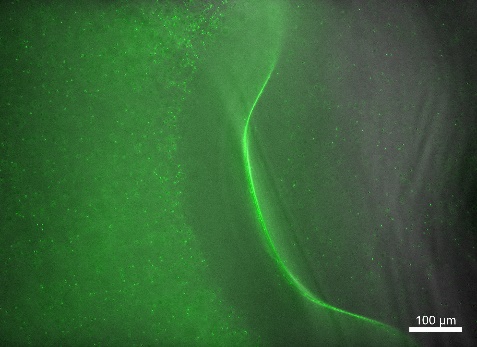


**gel**

**liquid**

**merged**

**fluorescence**

**brightfield**

**0.7 kPa PEG**

**1.5 kPa PEG**


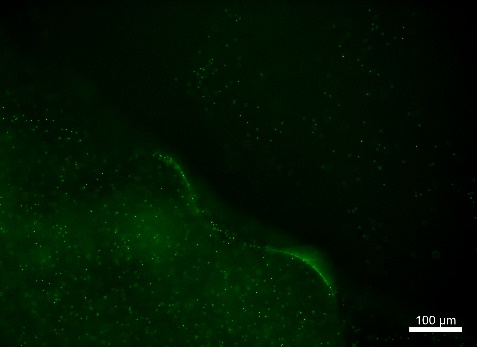

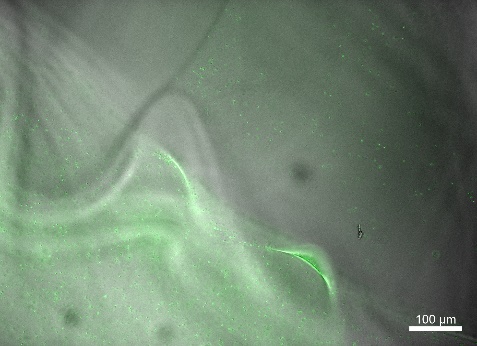

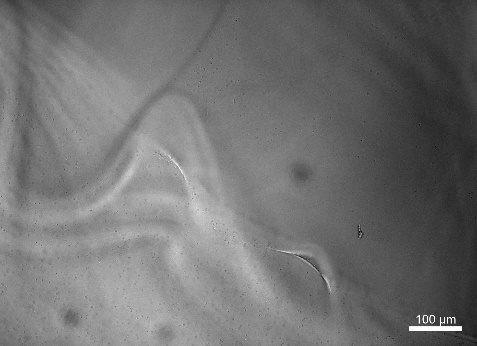


**gel**

**liquid**

**A**


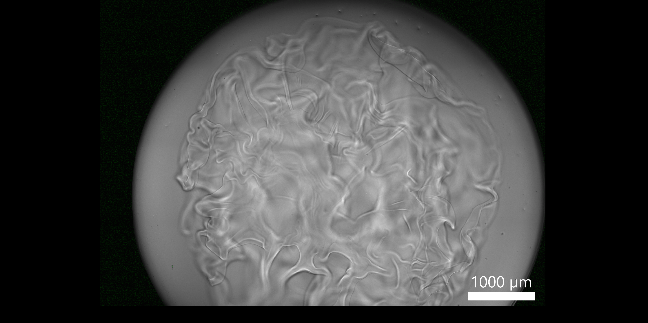


**brightfield**


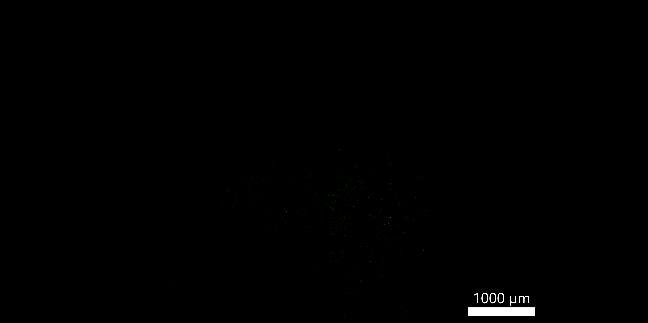


**fluorescence**

**B**

**Figure S2.** The presence of micropores in the PEG hydrogels was further confirmed through the infiltration of 1 µm fluorescent microparticles. Hydrogels were prepared as described in Section 2.5, without adding the microparticles in the crosslinker. After complete gelation, PBS containing fluorescent microparticles was added on top of the gels. Following overnight incubation, imaging was performed at the mid-section along the z-axis (avoiding the top and bottom surfaces) and at the interface of the hydrogel and the PBS. Panel A shows fluorescent microparticles within the gel region, confirming particle infiltration and providing additional evidence for the existence of micron-sized pores within the PEG hydrogels. The gel-liquid interface is indicated with orange dashed lines. This boundary was identified using a lower magnification imaging (Panel B, left), which enabled clear distinction between gel and liquid regions. Higher magnification was then used to image specific regions near the interface. The fluorescence image in Panel B (right) shows negligible background fluorescence in the gel prior to microparticle addition.


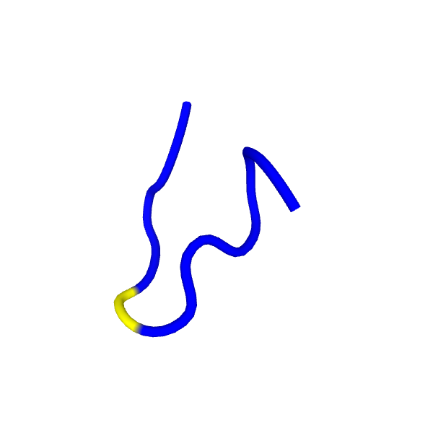

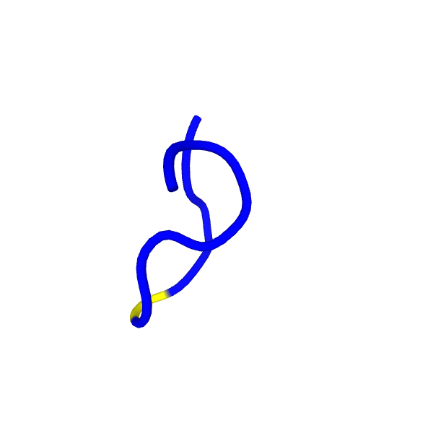

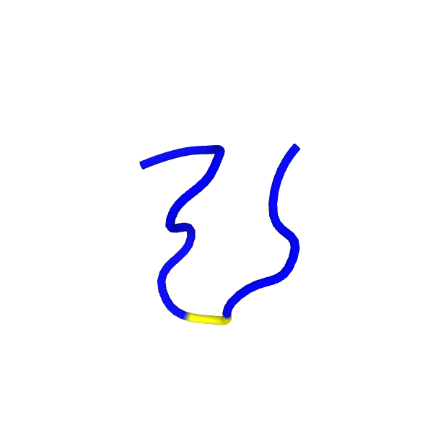

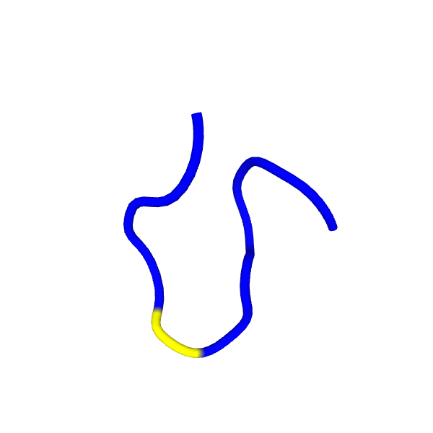


**Figure S3.** Representative cartoons of the peptide crosslinker modelled by PEP-FOLD 3 displayed at four different viewing angles. The proline amino acid at the loop is highlighted in yellow as a reference for the different viewing angles.

**
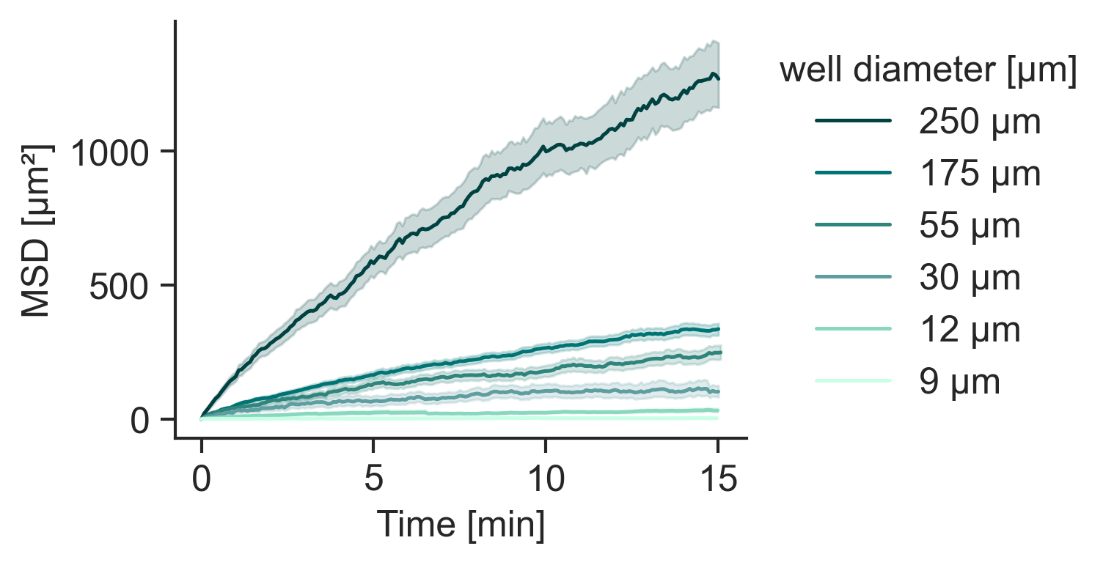
**

**Figure S4.** Mean-squared displacement curves of 1 µm particle diffusing in wells with varying diameters.


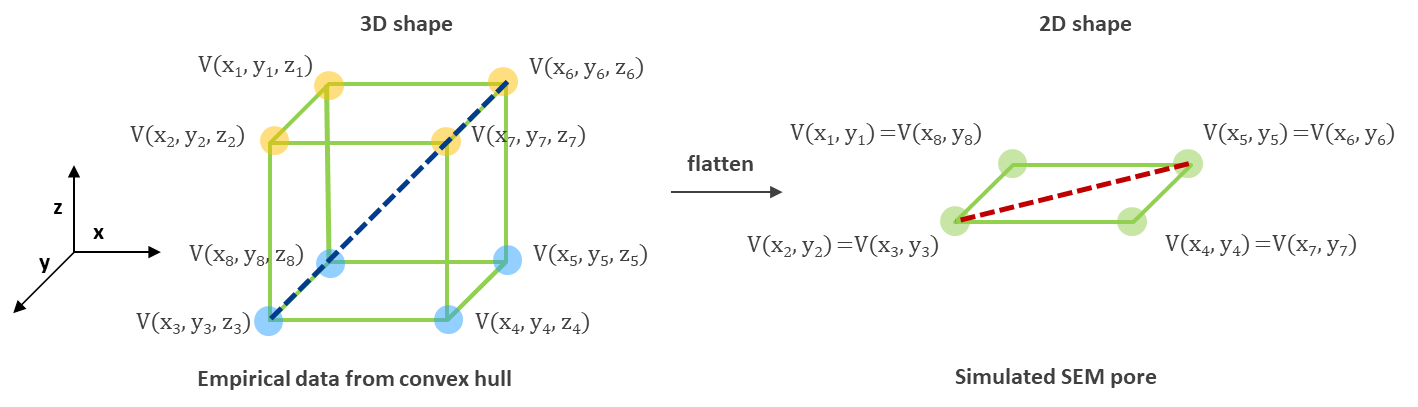


**Figure S5.** Illustration of principle to simulate SEM pore from 3D convex hull data. Here, we used a perfect cube as an example to simplify the illustration. The principle can be applied to any geometrical solid. V(x_i_, y_i_, z_i_) refers to vertex coordinates that are highlighted in yellow, blue and green circles. The dashed blue line refers to the longest diameter in the cube (3D) while the dashed red line refers to the longest diameter in the parallelogram (2D).
